# Development of the Placenta in the Fallopian Tube in Three Successive Pregnancies, Followed by Death from Rupture of the Uterus in a Succeeding Pregnancy

**Published:** 1853-11

**Authors:** 


### ECLECTIC DEPARTMENT,

### AND SPIRIT OF THE MEDICAL PERIODICAL PRESS.

*Development of the Placenta in the Fallopian Tube in three successive Pregnancies, followed by death from rupture of the Uterus in a succeeding Pregnancy.* By C. N. Andrews, M. D. Read before the Illinois State Medical Society.

Mrs. H. S. G., aged twenty-six, above medium height, of slender form but good organization, the mother of one living child three years of age, the result of her first pregnancy, suffered abortion on the 24th of March, 1845, when near the end of the third month of pregnancy; and also again on the 13th of November of the same year, at a somewhat earlier period. Conception again took place in the month of March following, (1846,) and on the 6th of the following November, I was sent for to attend her accouchment. In the early part of the afternoon she had been attacked with labor pains, and when I arrived the *os uteri* was considerably dilated and the membranes protruding. Labor progressed favorably, and ten minutes before 7 o'clock she was delivered of a small female child. The patient, at the delivery of the child, was in a kneeling posture, on the floor, and leaning forward and supporting herself in an arm-chair. A sudden and tremendous gush of blood immediately followed the birth of the child. The patient was placed in a supine position, and the ordinary measures to excite contraction of the uterus and to arrest hemorrhage from this organ were promptly used, but without success. Without much delay I searched for the placenta by tracing the cord with the index finger of my right hand, but it could not thus be reached. Slight traction upon the cord conveyed to the hand a sensation of firmness. I soon found my patient sinking so rapidly that I resolved on removing the contents of the uterus at once. I commenced by tracing the cord with the extended finger of my right hand, and continuing the search in this way, I found the placenta adhering by about one-third of its extent, to the right side of the uterus, near the fundus, the remaining portion floating with the membranes in fluid blood and coagula. I extended my hand under and posterior to the adhering portion and commenced detaching in an anterior direction, but I had not proceeded far in this way before I met with great resistance. I then drew my hand to the anterior side and commenced detaching in a posterior direction, but soon met with the same resistance as before; I then passed the end of my index finger back and forth around the adhering portion, and broke through several adhering filaments, but there was still adhering a portion of placenta of circular form, as large as I could span with my thumb and index finger, which no ordinary effort could detach. I tried to separate it from the uterus by tortion, but without success; also by traction, but this only drew the wall of the uterus into its own cavity which was not yet much reduced by contraction. Continuing my efforts, I grasped the placenta and by traction upon it drew, as I had previously done, the wall of the uterus into its own cavity, and then, with the parts in this condition, I passed the point of my index finger closely around the adhering portion, and felt a sharp circular ridge or projection surrounding it, which appeared like the margin of an opening from the uterine cavity. At this moment it occurred to me that a portion of the placenta was in the Fallopian tube. After a moment's reflection I commenced removing the detached floating portion of placenta by cutting it through with my thumb nail close to the uterine wall, supporting it on the opposite side with my index finger. This I did not accomplish without considerable difficulty, owing to the placenta being very dense and partly membranous at the point of division. After clearing the uterus the patient was placed in bed. She was very feeble; pulse 120, small and weak, had nausea, and vomited profusely. Stimulants were administered and she soon revived. At 9½ o'clock I left her for the night. She continued to improve, and nothing worthy of note occurred till on the 11th, when I was sent for in great haste. On entering her room I found all within much alarmed, and was told, on inquiring the cause, that "Mrs. G.'s womb had come down into the world." The patient was in great distress of mind and lay pressing both hands upon her vulva with a napkin interposed. I proceeded to explore the vagina in which I found a fleshy snbstance, slightly protruding externally and reaching the *os uteri*, which I removed at once and without difficulty. I learned, on inquiring, that on the previous evening the patient had been attacked with after-pains, which continued with occasional intermissions till a short time previous to my being sent for, when, being at stool, she felt something descend into the vagina, and her pains ceased.

The substance which I removed from the vagina was evidently a portion of placenta, and, without a show of doubt, in my mind, was that portion which I had left in the Fallopian tube and which had there remained till incipient putrefaction had destroyed its intimate connection with the parts containing it, when these parts, by their natural contraction, were competent to expel it. It possessed the usual appearances of placenta, except its increased density. It was of conical shape, about three and a half inches in length, and three and three-fourths in circumference at its base, and the cavity of the amnion extended quite to its apex, so that I was enabled to draw it on my finger as I would the finger of a glove. One side of this cavity was wholly membranous and lay pressed in longitudinal folds, in a groove-like depression, running the whole length of the fleshy portion, which depression formed its other side. The lining surface of this groove-like depression was smooth and membranous, and answered to the fetal surface of the placenta. The balance of surface of the fleshy or parenchymatous portion, which made up at least five-sixths of its circumference, was rough and granular, and answered to its uterine surface. The whole parenchymatous portion was so peculiarly firm and elastic that I could not cause it to maintain any other shape than the one I have described, without using an amount of force sufficient to break it down.

I saw the patient again on the 25th, when she had nearly recovered her usual health.

In the month of March, 1847, this woman again became pregnant, and on the 3d of December following, she was attacked with labor pains, and at 9 o'clock, P. M., of that day, I was called in attendance. When I arrived she was far advanced in labor, and in the course of half an hour the child was delivered. Profuse hemorrhage again immediately followed, which I was unable to check, except by such means as I had lastly resorted to at her previous confinement. The same signs of an adherent placenta were present, and, without delay, I introduced my hand into the cavity of the uterus and found that it was again adhering, and to the same part of the uterine cavity, to which I had found it adhering on the previous occasion. By making a minute examination I found about one-half of the placenta dipping into the Fallopian tube, the orifice of which appeared about twice as large as on the previous occasion. The same measures for removing the placenta were resorted to, as before, and with like success, except in this instance the increased size of the tubal orifice enabled me to remove a part of the invested portion. Moderate hemorrhage continued for some hours, but finally yielded. She discharged, *per vaginam*, for several days, putrid substances which were evidently the portions of placenta which I had not succeeded in removing. She regained her health gradually and without any unpleasant occurrences.

In the month of April, 1848, Mrs. G. again became pregnant, and her previous childbed history led me to watch her with much solicitude and interest. I was apprehensive of an extra-uterine or tubal pregnancy. Her becoming pregnant again, caused her great distress of mind, which amounted almost to despair. I was requested by her husband to visit her frequently and do what I could to quiet her fearful forebodings, and to satisfy myself, by making examinations, from time to time, in regard to anticipated difficulties. She having passed the second month without any untoward circumstances occurring, and the usual symptoms of *uterine* pregnancy presenting themselves, I concluded I should not realize my worst apprehension. Toward the end of the seventh month my attention was called to a tumor in the right side of the abdomen, above the anterior superior spinous process of the ilium, which appeared to be situated in, or attached to the parietes of the gravid uterus. A careful examination of this tumor convinced me that it was the placenta, and that it was a third time developed in the Fallopian tube. This tumor moved as the uterus moved when the patient turned from one side to the other in bed. A *souffle* of the character of the uterine or placental was distinctly heard in it, and it pulsated with a fine, vibrating and lengthened pulsation.

The patient had discovered this tumor, her attention having been called to it by severe pain in that part of the abdomen; and the nature of her previous childbed difficulties having, by her request, been explained to her, she very soon assigned a cause for it.

On the 4th of January, 1849, I was again called to attend her, she then being about eight months advanced in pregnancy. When I arrived her labor pains were severe, the *os uteri* dilated, the membranes protruding, and in a short time she was delivered of a dead child in the incipient stages of putrefaction. As on previous occasions, hemorrhage immediately followed to an alarming degree. I at once introduced my hand into the uterus, which was relaxed and distended with fluid blood and coagula, the fundus nearly reaching the epigastrium. I searched for the placenta, but nothing except the umbilical cord and loose floating membranes could be found in the uterine cavity. The presence of my hand in the uterus excited only slight contraction. I traced the cord which led to an opening in the right wall of the uterus into which it penetrated and from which, surrounding the cord, hung the fetal membranes. Into this opening, the margin of which was abrupt and unyielding, I readily introduced my thumb and two fingers half their length. Having done this I placed my left hand on the integuments of the right side of the abdomen, when I could feel, directly over or external to the points of my thumb and fingers introduced into the opening in the right uterine wall, a tumor corresponding with that which both the patient and myself had observed some weeks previously, except that as the fetus no longer crowded it against the abdominal wall, it required to be supported by my right hand and pressed outwardly in order to be distinctly felt. By grasping this tumor with my left hand, and thus holding it as firm as possible to facilitate the labor of my right, I was enabled, through the opening in the wall of the uterus, to break down the placenta and remove the larger portion of it a shred at a time. As the operation progressed, the pressure and counterpressure between my right hand and left rendered it certain that what I removed with my right hand came from within the grasp of my left; I also observed that in proportion as the placenta was removed the tumor softened and yielded in size.

So far as I could judge of the condition of the tube from the sense of touch, it appeared comparatively thin and not much if at all disposed to contract. It yielded freely to the movements of my fingers in any direction, and as I removed the placenta from it, it collapsed and felt more like a portion of large intestine than anything else I could compare it to, and thus presented a striking contrast with the thick, fleshy walls of the uterus and the rigid abrupt tubal orifice.

After removing as much of the placenta as the circumstances seemed to admit or require, and clearing the uterus, hemorrhage continued to a considerable extent for an hour or more, but the patient did not appear as much reduced as she had on either of the previous occasions on which I had attended her. She continued for a number of days to discharge small portions of putrid placenta, but no unfavorable occurrences followed, and she recovered rapidly, more so than after her previous confinement.

In consequence of a change of residence I lost sight of this patient, and heard nothing further from her till in the fall of 1851, when I learned accidentally, and in a very indirect way, that she was dead. For some time I could not learn when or at what place she had died. I addressed letters of inquiry to several persons residing in the state of New York, near where she had formerly resided, and at length I ascertained that she had died at Port

Byron, in that state. I continued my inquiries by addressing the physicians of that place, and from Drs. Button and Eldridge, who there reside, and from a brother of the patient who has recently removed to Rockford, I have learned the following particulars relating to her decease: On the 22d of June, 1851, when somewhere in the seventh month of pregnancy, while walking in the garden, she suddenly felt herself somewhat indisposed, and went into the house, when it soon became evident that she was in labor. Her pains had not continued long when she felt a sense of giving way or tearing in the abdomen, attended with great distress, which was immediately followed by pallor, great prostration, and a sense of impending death, and in an hour and a quarter she was a corpse.

A *post-mortem* examination was made the next day "*in great haste, and merely to ascertain the immediate cause of death,*" which was found to be "*a rupture in the right side of the uterus, caused, apparently, by chronic ulceration.*"

I regret, exceedingly, that a more particular examination could not have been made, and the exact relative situation of the rupture, the seat of the "*apparent ulceration,*" the place of attachment of the placenta, and the condition of the right Fallopian tube, noted in detail. It, however, seems to me very probable that the rupture was consequent upon disease produced by the repeated development of the placenta in the tube, together with some mechanical injury that might have been done by my repeated efforts to remove from that organ its abnormal contents at her previous confinements.

After somewhat diligent search, I have been able to find but four cases of development of the placenta in the Fallopian tube on record. D'Outrepont, Riecke, Pagan, and John Bell, have respectively noticed cases. The case of Mrs. G. confirms the deductions which have been drawn from these cases, to wit: that such development of the placenta predisposes to abortion or premature labor, and that it is uniformly attended with uterine hemorrhage.

There are two other questions of practical importance connected with Mrs. G.'s case: firstly, was the death of the fetus at her confinement on the 4th of January, 1849, caused by the placenta being wholly in the tube and a consequent interruption of the circulation through the cord and placenta? Secondly, I observed at her first and second confinements, when the placenta was only partially in the tube, that the hemorrhage was greatly lessened by detaching the placenta completely up to the margin of the opening of the tube, and having noticed this in the first instance, it influenced my subsequent practice. In her third confinement, when the placenta was wholly in the tube, the hemorrhage was not as sudden and profuse but continued longer than it had done previously, and my efforts to remove that organ did not have so much influence upon it.

My reflections upon these cases have led me to ask, in conclusion, this question: Is not an adherent placenta, and the usual coexisting hemorrhage, a frequent result of partial development of that organ in the tube; some of its roots, if I may so speak, being there planted, during the passage of the ovum, which there continue to vegetate?—*North Western Med. and Surg. Journal.*

